# The 6:1 short stitch SL-WL-ratio: short term closure results of transverse and midline incisions in elective and emergency operations

**DOI:** 10.1007/s10029-023-02927-4

**Published:** 2024-01-29

**Authors:** M. Golling, V. Breul, Z. Zielska, P. Baumann

**Affiliations:** 1 Diakonieklinikum Schwäbisch Hall, Schwäbisch Hall, Germany; 2 BBraun, Tuttlingen, Germany

**Keywords:** Non randomized, Controlled trial, Human cohort study, Laparotomy, Fascial closure technique, Short stitches, Small bites, 6:1 suture -/wound length (SL/WL) ratio, Median/midline and transverse incision, Elective and emergency surgery

## Abstract

**Aim:**

To analyze laparotomy closure quality (suture/wound length ratio; SL/WL) and short term complications (surgical site occurrence; SSO) of conventional midline and transverse abdominal incisions in elective and emergency laparotomies with a longterm, absorbent, elastic suture material.

**Method:**

Prospective, monocentric, non-randomized, controlled cohort study on short stitches with a longterm resorbable, elastic suture (poly-4-hydroxybutyrate, [p-4OHB]) aiming at a 6:1 SL/WL-ratio in midline and transverse, primary and secondary laparotomies for elective and emergency surgeries.

**Results:**

We included 351 patients (♂: 208; ♀: 143) with midline (*n* = 194), transverse (*n* = 103), and a combined midline/transverse L-shaped (*n* = 54) incisions. There was no quality difference in short stitches between elective (*n* = 296) and emergency (*n* = 55) operations. Average SL/WL-ratio was significantly higher for midline than transverse incisions (6.62 ± 2.5 vs 4.3 ± 1.51, *p* < 0.001). Results in the first 150 patients showed a reduced SL/WL-ratio to the following 200 suture closures (SL/WL-ratio: 5.64 ± 2.5 vs 6.1 ± 2.3; *p* < 0.001). SL/WL-ratio varied insignificantly among the six surgeons participating while results were steadily improving over time.

Clinically, superficial surgical site infections (SSI, CDC-A1/2) were encountered in 8%, while 4,3% were related to intraabdominal complications (CDC-A3). An abdominal wall dehiscence (AWD) occurred in 22/351 patients (6,3%)—twice as common in emergency than elective surgery (12,7 vs 5,1%)—necessitating an abdominal revision in 86,3% of cases.

**Conclusion:**

We could show that a short stitch 6:1 SL/WL-ratio with a 2–0 single, ultra-long term, absorbent, elastic suture material can be performed in only 43% of cases (85% > 4:1 SL/WL-ratio), significantly better in midline than transverse incisions. Transverse incisions should preferably be closed in two layers to achieve a sufficient SL/WL-ratio equivalent to the median incision.

**Clinical Trials.gov Identifier:**

NCT01938222.

## Introduction

Fascial suture technique is a delicate topic. On the one hand considered irrelevant by most surgeons, as was recently shown by a questionare, showing only a 35% [1] compliance to the gold standard (> 4:1 suture / wound length (SL/WL)-ratio) [2]. On the other hand, the taking over of laparoscopy over laparotomy even in high risk operations (smaller incisions with a lower hernia rate [3] and the anticipated low morbidity and near zero mortality when non-compliant may have added to the reluctance to improve closure technique. Nevertheless visceral surgeons do at least claim to perform the short stitch > 4:1 suture / wound length (SL/WL) ratio [4]. Relevance to this aspect is highlighted by the fact that the quality of the fascial suture may have an impact on surgical site infection (SSI) [5, 6], while definitely has shown to reduce the rate of burst abdomen and incisional hernia [6, 7].

Literature dates back to the 70’s and 80’s [8], when single stay sutures evolved from  a 2:1to  a 4:1 and eventually a continuous > 4:1 SL/WL-ratio [9]. Even a 6:1 SL/WL-ratio was introduced and resulted from a commonly performed whole stitch suture with large HR40 + needles through muscle and fascia (thickness ≈ 1 cm, respecting a stitch width > 1 cm and a (SI) of 1 cm. The widely used 0-loop suture insured strength and doubled suture material [10].

Meanwhile biomechanical and perfusional studies further added evidence that minimising trauma and reducing suture tension to the fascia and muscle are essential for reliable laparotomy closures [11–13].

Two decades ago, it has been shown by Israelsson et al., that a suture-to-wound-length (SL/WL) ratio > 4:1 will reduce the likelihood of wound infection and incisional hernia [5] while and thinner single sutures can provide similar, likely even better results than the traditional strong loop [14]. The same group implied in experimental studies that incorporating the short stitch technique on top will further reduce the incidence of incisional hernia rate [14]. Thus the ≥ 4:1 SL/WL ratio with the short stitch became the gold standard in fascial wound closure [2].

This was underlined in recent large prospective randomized trials with 2–0 polydioxanone [PDS] and p-4OHB-threads focusing on midline incisions. In the STITCH —trial [PDS], the 1 year incisional hernia rate was reduced from 21 to 13% [15] and recently the ESTOIH-trial [p-4OHB] could show that the short stitch technique may reduce the incisional hernia rate even further [16].

Aim of our’6:1 Short Stitch MonoMax®-trial ‘ was a combined innovative effort of using an elastic, extra-long-term, absorbent, monofilament suture material (poly 4-hydroxy-butyrate, [p-4OHB]) in *midline* and *transverse* incisions in *elective* and *emergency* surgical procedures reflecting fascial closure under real life conditions.

## Material and methods

The design, no of participants and statistical evaluation have been desciped in the trial protocol (Clinical Trials.gov Identifier: NCT01938222) and previously presented as updates in various European Hernia Society (EHS) conferences between 2018 and 2022. Our protocol obeyed the guidelines for reporting observational studies (STROBE) [17]. The surgical procedures carried out were following standard operating procedures and reflect the profile of a tertiary surgical center being academically linked to the university of Heidelberg as a teaching hospital. Participating surgeons (n = 6) were trained in domo and in situ and taught accordingly by the author (MG) who introduced the ‘short stitch’ and watched training videos of the parallel recruiting prospective, randomized trial (ESTOIH).

### Inclusion/exclusion criteria

We included patient data of 351 adult patients, planned for elective and emergency surgery aged ≥ 18 years (American Society of Anaesthesiologists (ASA) group I-IV), frequently high risk patients (stomach, liver, pancreas surgery) requiring midline and/or transverse incisions. Patient data included underlying disease and additional risk factors (pulumonary, cardiovascular, diabetes, arteriosclerosis, renal function etc.). All participants gave written informed consent. Ethical approval for this trial was obtained from the Ethics committee of the University of Heidelberg. Following the operation, all complications were recorded and analyzed prospectively. Pregnant women, patients with severe neurological and psychiatric disease and lack of compliance were excluded.

#### Surgical technique

The protocol for the suture technique was published previously in detail [18]. In brief, we counted the no. of stitches, measured the wound length, the incorporated thread to calculate the suture to wound length ratio (SL/WL-ratio) and could then calculate the average lateral stitch distance (LSD) and the interval of the stitches (SI) which may vary substantially according to the technique [5, 16, 18]. Please note: Due to various reasons, the first 50 sutures (documentation) were insufficient and had to be discarded altogether. After these shortcomings were addressed, we restarted the trial and decided to a) only include a limited amount of senior surgeons (*n* = 6, senior registrar level or above) and b) make sure that the surgeons themselves will be held accountable for sufficient documentation at the end of the operation (assisted by the documenting nurse).

#### Material

In all patients, an elastic, extra-long term, absorbent, monofilament suture manufactured from poly-4-hydroxybutyrate [p-4OHB] (MonoMax®, BBraun Surgical, S.A., Rubi, Spain) was used for closure of the fascia in an intended 6:1 SL/WL ratio. The single 2–0 thread length was 150 cm, armed with a 26 HR sized needle.

#### Protocol

Case report forms (CRF) were filled out by two junior surgeons (Z.Z., S.F.), not technically involved in the suture technique and later documented in the internet-based data file provided by BBraun. Results were anonymised by a case no. which could only be reallocated to the patient at the center side.

#### Outcome measures

According to the study protocol, the primary outcome measures were the surgical site infection rate (SSI) until day of discharge, according to the CDC (Centre of Disease Control and Prevention) classification [19]. Secondary outcome measures were reoperation rate due to burst abdomen, wound healing complications, length of postoperative stay and special reference to the suture material handling/ ergonomics (tissue drag, elasticity, knot security, knot pull tensile strength, knot run-down).

#### Statistics

All statistical analyses were done using SAS software version 9.4 (SAS Institute Inc., Cary, NC, USA). Multiple logistic regression models were calculated for earlier (< 150) and later (151–351) closures, midline and transverse incision and elective and emergency surgery. Backward elimination method was used for model reduction. The final models were limited to those three factors, age, gender, BMI and any pre-defined risk with a *p* value < 0.05. Experience of the surgeons were assessed after 50 and 150 operations indicating performance stitch quality over time while participating surgeons (no.1–6) were analyzed individually with respect to suture performance. Endpoints are presented as frequencies and rates; 95% confidence intervals are given when appropriate. The Chi-square test was used for rate comparison. Statistical significance was defined as a *p* value < 0.05 for the primary outcome. 95% prediction ellipses were used for visualization of scatter plot distribution.

## Results

### Patient and suture demographics (Table 1, Fig. 1)

**Table 1:** Demographics of the patient cohort (*n* = 351) included in the ‘Short Stitch 6:1 MonoMax trial’. Groups were assigned to elective and emergency surgery and according to experience (early operations: 1–150 and later operative stage: 151–351) during the trial

Patient clientel	Total	Indication for surgery			Patient groups (early / late)		
Subgroups	Std.dev. (%)	Elective ~	Emergency ~	*p*	no. 1–150	no. 151–351	*p*
Patients (*n*)	351 (100%)	296 (84.3%)	55 (15.7%)	< 0.05	150 (42.7%)	201 (47.3%)	n.s
Age	66.8 ± 13.3	67.2 ± 12.8	64.7 ± 16	n.s	66.9 ± 13.2	66.7 ± 13.4	n.s
♂/♀(*n*)	208 / 143	177 / 119	31 / 24	n.s	95 / 55	113 / 88	n.s
Duration of surgery (min)	163 ± 78	171 ± 80	120 ± 50	< 0.05	180 ± 80	151 ± 75	n.s
Incision length (cm)	32 ± 17	34 ± 17	25 ± 11	< 0.05	31 ± 16	33 ± 17	n.s
ASA 1 ASA 2 ASA 3 ASA 4	25 (7.1%) 146 (41.6%) 156 (44.4%) 24 (6.8%)	19 (6.4%) 130 (43.9%) 131 (44.3%) 16 (5.4%)	6 (10.9%) 16 (29%) 25 (45.5%) 8 (14.5%)	n.s n.s n.s < 0.05	7 (4.6%) 58 (38.6%) 74 (49.3%) 11 (7.3%)	18 (9%) 88 (43.8%) 82 (40.7%) 13 (6.5%)	n.s n.s n.s n.s
Duration on ICU (days)	4.1 ± 7.0	4.1 ± 7.0	4.1 ± 7.0	n.s	4.1 ± 7.0	4.1 ± 7.0	n.s
Carcinoma	195 (55.6%)	175 (59.1%)	35 (63.6%)	n.s	56 (37.3%)	139 (69.1%)	n.s
Hypertension	161 (45.9%)	146 (49.3%)	15 (27.3%)	n.s	84 (56%)	77 (38.3%)	n.s
Alcohol abuse	121 (34.5%)	103 (34.8%)	18 (32.7%)	n.s	58 (38.7%)	63 (31.3%)	n.s
Smoker (prev./actual)	50/45 (27%)	45/35 (27%)	8/10 (32.7%)	n.s	21/19 (26.7%)	29/26 (27.4%)	n.s
Diabetes (NIDDM/IDDM)	58/11 (19.6%)	54/9 (21.3%)	4/2 (10.9%)	< 0.05	34/2 (24%)	24/9 (16.4%)	n.s
Obesity (BMI > 30)	61 (17.4%)	35 (11.8%)	10 (18.2%)	n.s	36 (24%)	25 (12.4%)	n.s
Coagulopathy	61 (17.4%)	48 (16.2%)	13 (23.6%)	n.s	40 (26.7%)	21 (10.4%)	n.s
Asthma / COPD	35 (10%)	30 (10.1%)	5 (9.1%)	n.s	17 (11.3%)	18 (8.9%)	n.s
Liver disease	34 (9.7%)	31 (10.5%)	3 (5.5%)	n.s	17 (11.3%)	17 (8.5%)	n.s
Peritonitis	26 (7.4%)	6 (2%)	20 (36.4%)	< 0.05	9 (6%)	17 (8.5%)	n.s
Psychiatric disease	23 (6.5%)	20 (6.8%)	3 (5.5%)	n.s	10 (6.7%)	13 (6.5%)	n.s
Renal insufficiency	22 (6.3%)	16 (5.4%)	6 (10.9%)	n.s	12 (8%)	10 (5%)	n.s
Mortality	17 (4,8%)	12 (4,1%)	5 (9,1%)	< 0.05	7 (4,6%)	10 (5%)	n.s

Differences were noted between the elective and emergency operations and marked significant (*p* < 0.05)

**Fig. 1:**
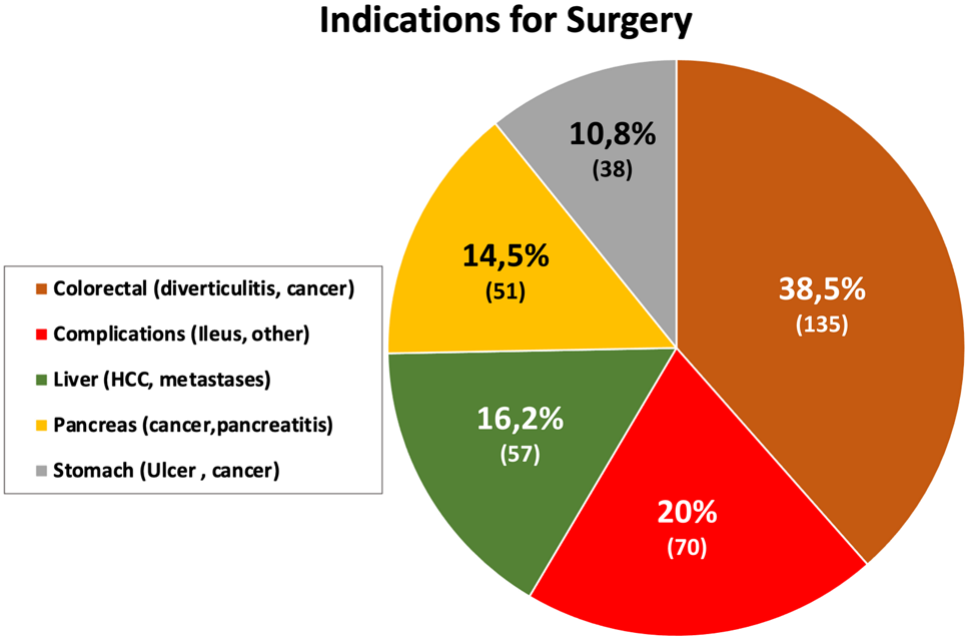
Patient cohort (*n* = 351) and indications for surgery with respect to organ allocation (colorectal, liver, pancreas, stomach) or other indications i.e., complications)

We included 351 predominantly male patients (♂: *n* = 208 (59.3%); ♀: *n* = 143 (40.7%). An adverse event occurred in 109 cases (31.1%), premature termination was implemented in 13/351 cases (3.7%). Since we perform the majority of our lower risk operations laparoscopically, the patient clientel recruited higher risk elective (*n* = 296, 84.3%) and emergency (*n* = 55, 15.7%) operations. Patients included involved all general and visceral operations from adhesions and bleedings (emergency) up to complex elective liver and pancreatic surgery (Fig. 1). In general, these procedures require open, conventional surgery, resulting in a significant morbidity (e.g., surgical site occurrence/ SSO), and mortality. On average, duration of surgery was quite long (163 ± 78 min) and differed significantly between the faster emergency (120 ± 50 min) and slower elective (171 ± 80 min, *p* < 0.001) operations. Complex elective liver and pancreas operations required longer incisions (*p* < 0.05). As a consequence, > 26% of patients eventually spent > 3 days (4.1 ± 7.0 days) on the ICU. The mortality rate of 4.8% reflects the comparatively high inclusion rate of ASA 3 (44.4%), ASA 4 (6.8%) cancer and emergency patients (Table 1).

### Incisions (Tables 2 and 3, Fig. 2)

**Table 2:** Presentation of quality performance indicators (SI, LSD and SL/WL-ratio) in the patient cohort (*n* = 351), subdivided into the midline, transverse, and hook (L)-shaped (combined midline and transverse) incision population

Performance indicators (no. of patients)	Patients overall (*n* = 351)	Midline (*n* = 194)	Transverse (*n* = 103)	Hook-L-shaped (*n* = 54)	***p***
Incision length (cm)		22.3 ± 6.0*	22.7 ± 9.9*	43.8 ± 11.7	< 0.001
stitch interval (SI)	0.42 ± 0.1	0.38 ± 0.1*	0.47 ± 0.1	0.44 ± 0.08*	< 0.001
lateral stitch distance (LSD)	0.59 ± 0.23	0.61 ± 0.26	0.54 ± 0.18*	0.62 ± 0.16	< 0.001
SL/WL-ratio	5.9 ± 2.4	6.6 ± 2.6	4.7 ± 1.7	5.8 ± 1.3	< 0.001

**Table 3:** Presentation of quality performance indicators (SI, LSD, SL/WL-ratio) in the cohort with respect to sutures (*n* = 509), subdivided into isolated midline and transverse layers (separate anterior/posterior fascial) and a distinct transverse all layer (combined anterior/posterior) sutures

Performance indicators (no. of sutures)	Sutures overall (*n* = 509)	Midline (*n* = 242)	Transverse (*n* = 117)	Transverse all layer (*n* = 33)	***p***
stitch interval (SI)	0.43 ± 0.11	0.39 ± 0.1	0.47 ± 0.1	0.39 ± 0.1	< 0.001
lat. stitch distance (LSD)	0.58 ± 0.22	0.63 ± 0.25	0.53 ± 0.18	0.63 ± 0.25	< 0.001
SL/WL-ratio	5.61 ± 2.29	6.59 ± 2.51	4.71 ± 1.63	6.58 ± 2.48	< 0.001

**Fig. 2:**
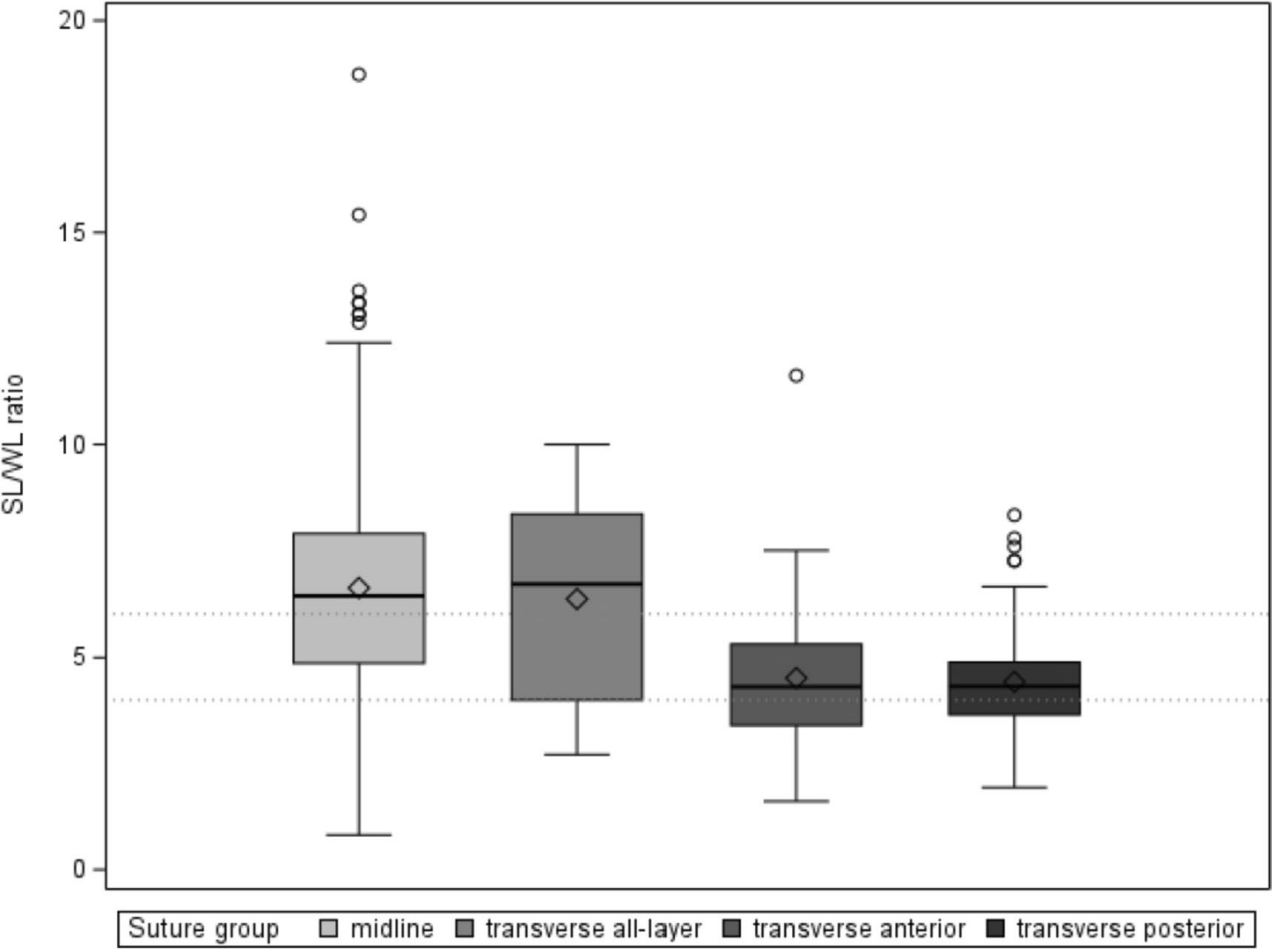
SL/WL-ratio (box plots showing median, 50% box, confidence interval and min./max value) for subgroups from the multivariate model (analysis related to sutures). SL/WL-ratio marked between groups (transverse anterior/posterior and transverse both/all layer and midline)

We included standard midline (*n* = 194; 55.3%), transverse (*n* = 103; 29.3%) and combined inverted L-shaped (or hook-like) incisions (*n* = 54; 15.4%), which involved a midline and right transverse closure. Incision length varied understandably. Almost all transverse incisions (*n* = 117) were closed separately, anterior and posterior fascial layers. Incisions for emergency cases were exclusively midline laparotomies (*n* = 55), while gastric and pancreatic operations were typically done via tranverse (subcostal) incisions. Liver operations (*n* = 54) were routinely performed with an inverted L-shaped (hook-like) incision (Table 2). On a patient basis, performance quality indicators SI, lateral stitch distance (LSD) and SL/WL) of L-shaped incisions are somewhat distorted as they involve midline and transverse incisions. For the comparison of suture quality, hook-L-incisional sutures were then integrated into the midline and transverse closures (Table 3).

Comparing midline and transverse suture quality, we could clearly show that performance indicators can be fulfilled following a midline incision, while failing in transverse sutures (Table 3). Despite fulfilling the > 4:1 gold standard in > 90% of midline closures and > 80% of transverse closures, a 6:1 SL/WL ratio can only consistently be achieved in a midline suture (midline: 6.6 ± 2.5 vs transverse: 4.7 ± 1.6, *p* < 0.001), mainly due to a lower SI (midline: 0.39 ± 0.6 vs transverse: 0.47 ± 0.13; *p* < 0.001) and larger lateral stitch distance (LSD) values (midline: 0.63 ± 0.25 vs transverse: 0.53 ± 0.18; *p* < 0.001). Overall, a 6:1 suture was achieved in ~ 70% of midline and ~ 25% of transverse incisions. In transverse closures there was no difference in quality performance indicators when comparing anterior and posterior fascia (Table 3), despite of a stronger anterior layer and the combined suture of transversus muscle and fascia. On the other hand, in the all-layer transverse sutures, a 6:1 SL/WL-ratio was similar to the midline incision (Table 3, Fig. 2). Despite a wider confidence interval compared to midline incisions, the transverse all layer and midline sutures commonly show a 6:1 SL/WL-ratio.

### Experience (Table 1, Fig. 3) 

**Fig. 3:**
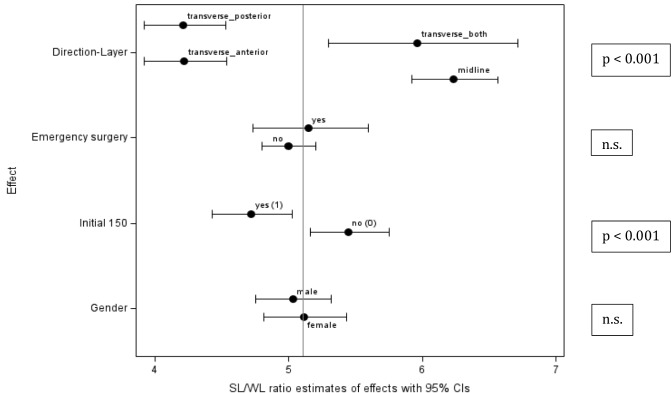
Effect of subgroups (Forest plots, means and confidence intervals) i.e., direction of fascial suture (layer), emergency vs elective, initial patients 1–150 vs 151–351 and gender. Taken from the multivariate model of SL/WL-ratio (analysis related to sutures (*n* = 509)). Significance in SL/WL-ratio marked between groups (transverse posterior/anterior vs transverse both/all layer and midline) and experience of surgeons (initial 150 vs next 200 patients)

To assess the technical experience for the 6:1 SL/WL-ratio plus short stitch technique, we could show that for the first 50 patients only 25% actually achieved a > 6:1 ratio, while 47% even failed to reach > 4:1 SL/WL-ratios (data not shown, presented at the EHS 2018). We then restarted and arbitrarily chose the first 150 operations to fix a first setpoint for the evaluation of the quality performance indicators. We could show a significant improvement in SI, LSD and SL/WL-ratio and—following an evaluation—aimed at improving the technique with the remaining 201 operations. From then on results substantially improved to 44% (> 6:1) and 87% > 4:1 SL/WL-ratios. Quality performance indicators could clearly show a highly significant improvement > 150 patients in SI (≤ 150: 0.38 ± 0.1 to > 151 0.44 ± 0.1; *p* < 0.001) and LSD (≤ 150: 0.51 ± 0.2 to > 151: 0.65 ± 0.23;* p* < 0.001) eventually resulting in a SL/WL-ratio of 6.1 ± 2.3 (> 151–351) coming from 5.6 ± 2.5 (1–151; *p* < 0.001, Fig. 3).

### Urgency (elective vs emergency) (Table 1, Fig. 3)

No signicant differences of quality performance indicators were noted between elective and emergency cases (LSD, SI, SL/WL-ratios). When comparing SL/WL quality performance, emergency parameters even slightly outperformed elective surgery which may be attributed to the more predominant midline incision in these conditions.

### Individual surgeons (Fig. 4)

**Fig. 4:**
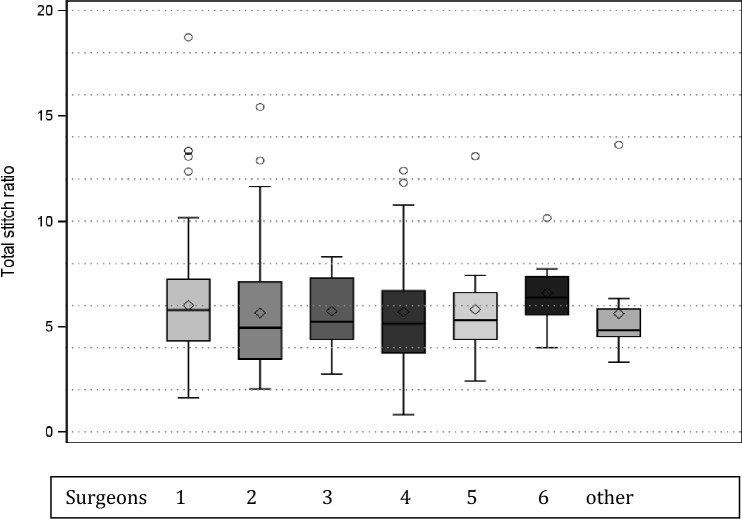
SL/WL-ratio in the surgeons (1–6 and others) involved, shown in absolute numbers of sutures [N] and a Forest plot with min/max-whiskers, mean/median/standard deviation, and 50% box (n.s.)

Individual surgeons achieved in 85% of sutures a > 4:1 SL/WL ratio closure, while an > 6:1 SL/WL ratio resulted in 43%. Despite some heterogenous performance was seen, the overall results showed no significance amongst surgeons. These difference in the early stages improved in all participants (data not shown) and eventually approximated each other.

### Surgical site occurrence (SSO) (Figs. 5 and 6)

**Fig. 5:**
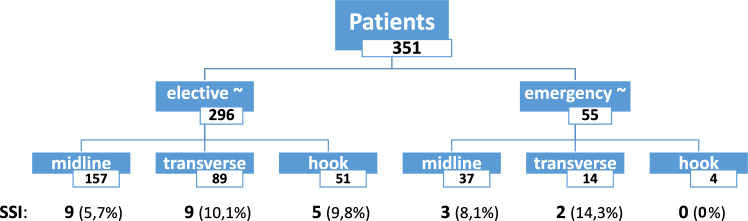
Patient cohort (*n* = 351) separated into urgency categories (elective / emergency) and incision (midline / transverse / combined hook-L-shaped) showing associated grade A1/2 CDC surgical site infections (SSI) in absolute numbers (*n* = 28) and relative values (%) in relation to the incision groups

**Fig. 6:**
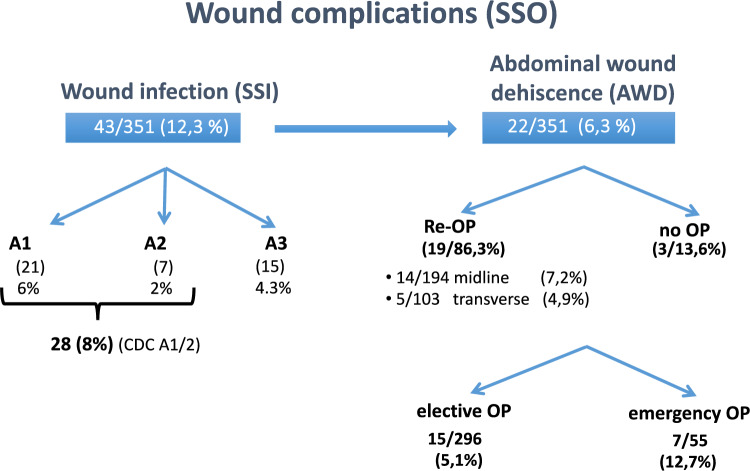
Wound complications (surgical site occurence / SSO) in the 6:1 short stitch MonoMax trial cohort (*n* = 351): Surgical site infection (SSI) were graded according to the CDC-Classification, only A1 and A2 infection contributing to infections potentially influenced by surgical technique

Beside the overall and individual technical performance, the primary objective was detecting early wound complications like SSI and wound dehiscence/burst abdomen. Wound infections (12.3%) occured in > 50% of the cases between day 3 and 8 (data not shown). We limited ourselves to the CDC grade A1 and two infections (cutaneous till fascial level) and left grade A3 (organ specific infection, leakage, anastomotic insufficiency) being irrelevant for assessment of fascial suture technique. We encounterd 8% SSI Grade A1/A2 infection, an abdominal wound dehiscence in 22/351 cases (6.3%), mostly seen in Grade A3 complications necessitating a reoperation in 86,3% of cases. Emergency operations result in a more than doubling of the AWD rate (12,7% vs 5,1%). While an AWD was almost all cases (21/22) associated with an SSI, only one patient had a ‘classical’ burst abdomen situation (on postoperative day 2) with ascitic leakage and evisceration (♂/68 yrs, hepatocellular carcinoma, liver resection, hepato-renal syndrome, ascites, low albumin etc.).

Comparing elective with emergency surgery and talking incision location into account, SSI are seen more commonly in transverse than midline incisions and are more common in extensive operations (liver, pancreas) and under emergency conditions (Fig. 5).

## Discussion

So far randomized studies involved in the short stitch technique focussed on the short (SSI, SSO) and longterm (hernia incidence after 1–3 years) complications or success rates vice versa. In almost all trials, a median/midline incision was chosen, likely because of a more standardized closure (straight line, universally more common) and fascial properties of the line alba (collagen structure, thickness, rigidity). Interestingly, the technical difficulties or adherence and complicance to protocol has rarely been shown [15, 16, 20, 21]. Recently a spanish study explicitly addressed this topic showing that only 52% of surgeons performed the required calculation (SL/WL-ratio), resulting in only 30,7% small bites and consequently a lower burst abdomen and incisional hernia rate (3,6 vs 12,1%) [22].

Our study focused explicitly on the overall and individual quality performance technique in *all kinds* of *incisions* (midline, transverse, L-shaped) and under *all kinds *of *conditions* (elective and emergency surgery) [8, 23]. Since most elective even larger oncological/ abdominal procedures are nowadays at least contemplated to be performed laparoscopically, our patient clientel here is regarded as a high risk cohort from the presenting but also underlying disease. This is underlined by the 51.2% ASA-3/4 group contributing to the mortality rate of 4.8%.

We know of three randomized-controlled trials investigating the effect of the short stitch suture technique using polydioxanone [PDS] as the suture material [6, 15, 24]. Recently the ESTIOH-trial using p-4OHB (same thread as in this study) as alternative suture material involved 425 patients showed a clear benefit by cutting in half the incisional hernia incidence (3.3% vs 6.4%) compared to conventional 4:1 SL/WL ratio [16].

All these randomized studies are impaired by the bias of exclusively including *midline* incisions in *elective* patients. We aimed at exceeding the current gold standard (> 4:1 SL/WL-ratio) by further increasing the SL/WL-ratio to > 6:1 plus integrating the short stitch technique simultaneously. Interestingly, the ESTOIH-trial with the identical suture material [p-4OHB] showed a substantially lower hernia rate at 1 year (short vs long stitch: 3.3% vs 6.4%) than previously published data with PDS-plus between 13 vs 21% [6, 15]. Technically, the short stitch group (ESTOIH) with a SL/WL-ratio of 5.3 ± 2.2 almost reached our targeted values and significantly exceeded the conventional 4:1 gold standard. Whether the material or the thread properties (elasticity) might have contributed to a lower hernia rate is speculative [16, 18]. We encountered a positive feedback from the surgeons handling the thread.

It was not surprising that quality performance indicators for midline incisions outplayed transverse incisions, since collagen structure, width and tensile strength of the linea alba will assure a better overall SL/WL ratio [11, 23]. The use of a small bore 26-needle and 2–0 thread has resulted in a better performance of the short stitch technique within the landing zone of the stitch and has established itself as superior to a more traumatising wide, fascia/muscle combined stitch with a 35–40 HR needle with a loop (ESTOIH). Nevertheless, the collagen fibre arrangements and the lower strength of the ventral and especially dorsal fascia of the rectus and the transverse abdominis muscle [25, 26] may have contributed to button holes in the fascia and muscle resulting in a significantly lower lateral stitch distance (shorter SL/WL-ratio).

Next to the technical assessment, our secondary objective was the observation of SSO (especically AWD) in the early postoperative phase [27]. We encountered a grade A1/2-SSI more commonly in transverse incisions and emergency operations clearly underscoring the relevance of the severity and urgency rather than access route and duration. An AWD (6.3%) was almost always associated with an SSI (21 out of 22 cases). Interestingly, even in CDC-A3 infections (leakage from an anastomosis or parenchymal resection surface), the clinical detection did not reveal itself through a wound dehiscence but rather the drainage. To our understanding, the 6:1 short stitch resulted in a fully functional separation of the abdominal compartment from the subcutaneous space.

Finally, it is worth mentioning that the short stitch might not always be better. A recent study found a higher rate of burst abdomen (4% vs 0%) in relaparotomy cases (RELAP study) when the short stitch was applied [28].

To our knowledge, we have for the 1^st^ time addressed a 6:1 SL/WL-ratio with a short stitch technique in midline and transverse incisions in elective and emergency conditions [8, 23]. Our technical analysis is clear: In midline laparotomies, we can—after a thorough technical assessment—perform a 6:1 short stitch SL/WL ratio in > 70% of cases, while this cannot be standardized for transverse incisions (< 25%). Thus, a seperate closure of the dorsal and ventral fascia is strongly advised.

Considering the current evidence, the authors would rate the surgeons’ devotedness (to fascial closure) and meticulous short stitch performance (proven by quality control) as the essential driving force for improvement of results and consequently reduction of SSOs for the future [20–22].
